# Machine learning modelling of blood lipid biomarkers in familial hypercholesterolaemia versus polygenic/environmental dyslipidaemia

**DOI:** 10.1038/s41598-021-83392-w

**Published:** 2021-02-15

**Authors:** Marta Correia, Eva Kagenaar, Daniël Bernardus van Schalkwijk, Mafalda Bourbon, Margarida Gama-Carvalho

**Affiliations:** 1 University of Lisboa, Faculty of Sciences, BioISI—Biosystems & Integrative Sciences Institute, Campo Grande, 1749-016 Lisboa, Portugal; 2 Amsterdam University College, Science Park 113, 1098 XG Amsterdam, The Netherlands; 3 National Institute of Health Doutor Ricardo Jorge, Padre Cruz Av., 1649-016 Lisboa, Portugal

**Keywords:** Machine learning, Biomarkers, Genetic testing, Dyslipidaemias

## Abstract

Familial hypercholesterolaemia increases circulating LDL-C levels and leads to premature cardiovascular disease when undiagnosed or untreated. Current guidelines support genetic testing in patients complying with clinical diagnostic criteria and cascade screening of their family members. However, most of hyperlipidaemic subjects do not present pathogenic variants in the known disease genes, and most likely suffer from polygenic hypercholesterolaemia, which translates into a relatively low yield of genetic screening programs. This study aims to identify new biomarkers and develop new approaches to improve the identification of individuals carrying monogenic causative variants. Using a machine-learning approach in a paediatric dataset of individuals, tested for disease causative genes and with an extended lipid profile, we developed new models able to classify familial hypercholesterolaemia patients with a much higher specificity than currently used methods. The best performing models incorporated parameters absent from the most common FH clinical criteria, namely apoB/apoA-I, TG/apoB and LDL1. These parameters were found to contribute to an improved identification of monogenic individuals. Furthermore, models using only TC and LDL-C levels presented a higher specificity of classification when compared to simple cut-offs. Our results can be applied towards the improvement of the yield of genetic screening programs and corresponding costs.

## Introduction

Dyslipidaemia is one of the major cardiovascular risk factors and it is commonly associated with increased levels of serum low-density lipoprotein cholesterol (LDL-C) and/or reduced levels of high-density lipoprotein cholesterol (HDL-C), as well as high levels of triglycerides^1,2^. Once serum LDL particles exceed a threshold concentration, atherogenesis—an inflammatory process that precedes atherosclerosis—is stimulated, eventually leading to the development of fatty lesions (i.e. atheromatous plaques) on the lumen surface of large- and intermediate-sized arteries^1,3^. As a silent condition, dyslipidaemia usually produces no symptoms until the unexpected occurrence of an acute cardiovascular event^1^.

In addition to being secondary to other disorders or having nutritional causes, dyslipidaemia can occur as a consequence of specific genetic defects^4^. Familial hypercholesterolaemia (FH), an autosomal dominant disorder, is the most common monogenic dyslipidaemia, with an estimated heterozygous prevalence of 1/250 worldwide^4,5^. FH increases circulating LDL-C mainly by affecting LDL receptor function, with undiagnosed and untreated subjects being at extremely high risk of premature cardiovascular disease (CVD)^3,6^. These dyslipidaemic subjects present the most severe phenotype and prompt and accurate diagnosis is essential for CVD prevention, allowing earlier and/or more aggressive therapeutic measures, which have been shown to be effective at reducing cardiovascular morbidity and mortality in both adults and children^6–8^.

Given the silent nature and prevalence of FH, current guidelines support the testing of genes encoding the low-density lipoprotein receptor (*LDLR*), apolipoprotein B (*APOB*), and proprotein convertase subtilisin/kexin 9 (*PCSK9*) in patients that comply with clinical diagnostic criteria, and cascade screening of their family members^9^. However, most hyperlipidaemic subjects do not have a monogenic defect^4,10^. Rather, their disease is most likely established through a polygenic genetic background, with a variable environmental contribution modulating the phenotypic expression^4,10^. Although the lipid profile of polygenic subjects is usually less severe than that of FH subjects regarding total cholesterol (TC) and LDL-C levels, the differences are often subtle enough to prevent an accurate distinction between the two conditions^3^. As a consequence, the yield of FH genetic screening programs is relatively low, assuming significant costs for patients and/or national health systems.

The Portuguese FH study (PFHS) has been performing a systematic characterisation of FH cases in Portugal since 1999 and includes extended lipid profiles for a large number of index patients^11^. Previous work using data from this study revealed that the approximately 60% of children that complied with the Simon Broome (SB) clinical criteria for FH were negative for mutations in the hallmark genes, most likely corresponding to cases of polygenic hypercholesterolaemia^12^. FH-positive subjects (FH+, carrying a pathogenic/likely pathogenic variant) showed higher concentration of atherogenic (i.e. LDL-C) and lower concentration of anti-atherogenic particles (i.e. HDL-C)^12^. In contrast, most of FH-negative subjects (FH−, no causative variant found) presented higher levels of triglycerides (TG), apolipoprotein C-II (apoC-II), apolipoprotein C-III (apoC-III), apolipoprotein E (ApoE), as well as higher frequency of overweight/obesity^12^. This suggests that the integrated analysis of multiple biomarkers could be used to create a model that can effectively discriminate between these two populations, improving the selection of patients for genetic screening. Furthermore, a better understanding of the lipid profiles of FH+ and FH− patients may shed further light on the molecular and genetic basis of polygenic hypercholesterolaemia, eventually leading to the identification of novel biomarkers and/or therapeutic targets.

In this work we used a machine learning approach to explore the paediatric subset of the PFHS 2018 dataset update (PFHS-ped) to develop novel models that can integrate data from multiple biomarkers and achieve a reliable discrimination between individuals. Our systematic exploration of available lipid parameters resulted in the development of several models that can robustly classify subjects into FH+ or FH− classes. Some of the models have parameters not routinely used in clinical practice but that are commercially available. Notwithstanding, models comprising only the standard lipid parameters used in the clinic also achieved a relatively good performance. Our results provide an approach for improving the yield of genetic screening programs while showing distinct biochemical backgrounds in monogenic and polygenic hypercholesterolaemia.

## Subjects and methods

### Patient selection, biochemical and clinical data

The work dataset—PFHS-ped—comprises a subset of 211 unrelated children (from 2 to 17 years old) from PFHS ^11^ that were not undergoing statin treatment at the time of referral and for which BMI and a basic set of lipid parameters were available (Supplementary Data S1). PFHS was approved by the National Institute of Health Ethic Committee and National Data Protection Commission. The study protocol conforms with the ethical guidelines of the 1964 Declaration of Helsinki and its later amendments. Written informed consent was obtained from parents or legal tutors. For this study, all data were fully anonymised before analysis.

The clinical criteria to be referred to the PFHS is the SB criteria. Between 2006 and 2011, patients with LDL-C or TC levels below the cut-offs established by SB criteria were admitted to the PFHS as long as TC was above the 95^th^ percentile for age and sex of the Portuguese population and a family history of hypercholesterolaemia was present, aiming at a better definition of the clinical criteria for FH in Portugal^11,13^. For the purposes of this study, we decided to include these individuals in the PFHS-ped dataset to increase the number of available cases. Thus, 68% of the 211 individuals in PFHS-ped fulfil the SB clinical criteria for FH^14^, while the rest present TC above the 95th percentile for their age and sex and a family history of hypercholesterolaemia^13^. All the individuals were subjected to molecular study, resulting in the classification of 88 individuals as FH+ and 123 as FH−, defined respectively by presence or absence of known FH causal variants in *LDLR*, *APOB* or *PCSK9* genes^13^.

Individuals presenting genetic variants of unknown significance according to the American College of Medical Genetics and Genomics guidelines^15^ were excluded from this study.

The PFHS-ped includes BMI, age and an extended characterization of lipid profiles, including quantification of small dense LDL (sdLDL), apolipoproteins (apo) A-I, A-II, B, C-II, C-III and E and a ‘Lipoprint’ profile measuring different subfractions of LDL-C (Table 1). The blood lipid profile was divided in three different levels: ‘Basic’, ‘Advanced’ and ‘Lipoprint’, for commonly determined, specialized and Lipoprint test lipid parameters, respectively (Table 1). Biochemical characterization of ‘Basic’ and ‘Advanced’ lipid profiles was performed as described before^12^. Briefly, fasting blood samples were collected from individuals and TC, direct LDL-C, HDL-C, TG, apoA-I, apoB, and lipoprotein (a) [Lp(a)] were determined for all individuals in a Cobas Integra 400 plus system (Roche) by enzymatic colorimetric and immunoturbidimetric methods. Serum levels of apoA-II, apoC-II, apoC-III, apoE, and sdLDL (sLDL-EX “SEIKEN” kit) were measured by direct quantification in an RX Daytona analyser (Randox Laboratories). The ‘Lipoprint’ profile was obtained using the ‘Lipoprint LDL subfractions test’ (Quantimetrix)^16^. This is a semiquantitative method that separates by polyacrylamide gel electrophoresis the different lipoprotein fractions as VLDL, IDL, LDL 1–7 subfractions (LDL subfractions 3–7 considered the sdLDL) and HDL^16–18^. For the purpose of this study, ratios that relate lipid parameters were calculated and included as additional variables to explore previous observations suggesting a differential contribution of TG and LDL metabolism and anti-atherogenic/pro-atherogenic factors to FH+ and FH− dyslipidaemic states (Table 1).

**Table 1:** Description of the biochemical parameters and ratios in each lipid profile—‘Basic’, ‘Advanced’ and ‘Lipoprint’.

Profile	Parameters		Units	Description
Basic	Biochemical	TC	mg/dl	Total cholesterol
		LDL-C		Low-density lipoprotein cholesterol
		HDL-C		High-density lipoprotein cholesterol
		TG		Triglycerides
		Lpa		Lipoprotein (a)
		ApoB		Apolipoprotein B
		ApoA-I		Apolipoprotein A-I
	Ratios	ApoB/ApoA-I	N/A	Anti-atherogenic vs pro-atherogenic ratio
		TG/ApoB		TG metabolism vs LDL metabolism ratio
		TC/HDL-C		Anti-atherogenic vs pro-atherogenic ratio
Advanced	Biochemical	ApoA-II	mg/dl	Apolipoprotein A-II
		ApoC-II		Apolipoprotein C-II
		ApoC-III		Apolipoprotein C-III
		ApoE		Apolipoprotein E
		sdLDL.Day		Small dense LDL
	Ratios	ApoC-II/ApoC-III	N/A	Anti-atherogenic vs pro-atherogenic ratio
		sdLDL/LDL-C		Most atherogenic LDL in total LDL-C
Lipoprint	Biochemical	VLDL	mg/dl	Very low-density lipoprotein
		MIDA		IDL fraction A
		MIDB		IDL fraction B
		MIDC		IDL fraction C
		LDL1		Buoyant (large) LDL fraction 1
		LDL2		Buoyant (large) LDL fraction 2
		HDL.Lipo		High-density lipoprotein
		sdLDL.Lipo		Small dense LDL (fractions 3 to 7)
		IDL		Intermediate-density lipoprotein
	Ratios	VLDL/IDL	N/A	TG metabolism vs LDL metabolism ratio
		VLDL/LDL-C		TG metabolism vs LDL metabolism ratio

*N/A* not applicable.

### Modelling and data analysis

The full description of modelling and data analysis methods is available as supplementary methods. Briefly, the *caret* package for machine learning^19^ was used to train classification models based on logistic regression, and a resampling scheme of three times cross validation was applied to estimate model accuracy. Accordingly, data was randomly divided in two sets of 60% and 40% of the subjects defining the training and the testing sets, respectively. The training set was used for model generation and the testing set was used for posterior validation. Models were ranked according to a set of statistical criteria (see supplementary methods) and the top 10 models are discussed in more detail in the context of the biology of hypercholesterolaemia.

## Results

### Definition of PFHS-ped data subsets for exploratory modelling of extended lipid profiles

**Figure 1:**
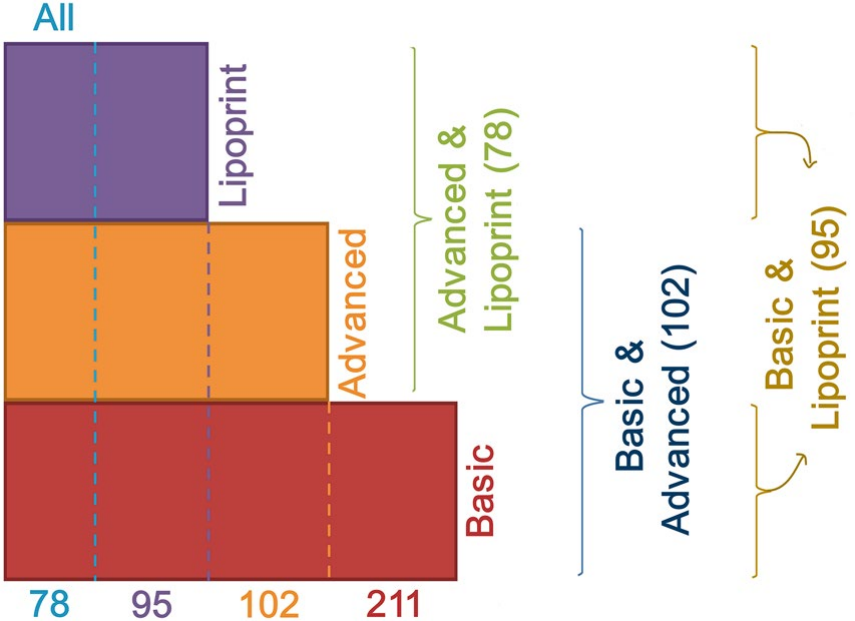
Data subsets used for model training. Figure shows how PFHS-ped was divided into smaller subsets, identified by a color-coded size (number of individuals) and name, according to the available biochemical parameters for each individual.

Given that the available information on lipid parameters varied between individuals and considering the three lipid profiles defined for this study—‘Basic’, ‘Advanced’, and ‘Lipoprint’, we began by establishing distinct data subsets regarding all the possible combinations of these profiles (Fig. 1). A detailed description of the seven data subsets is available as supplementary data (Supplementary Tables S1 and S2). As depicted in Fig. 1, the number of individuals across subsets varies between 78 and 211. Although relatively small, these numbers have been previously used in conjugation with machine learning approaches to derive valuable insights into complex biological problems^20–23^. We therefore set out to systematically search for the best model to discriminate between FH+ and FH− individuals using these different combinations of lipid parameters.

### Systematic training of models to distinguish FH+ and FH− subjects using extended lipid profiles

We began by training models using all available parameters in each subset. These ‘pilot models’ provided a rough overview of the behaviour of the different parameters in our data subsets but presented a very low performance as assessed by their sensitivity and specificity values (Supplementary Data S2). This suggested an overfitting problem, which we attempted to correct through the use of three common methods to reduce the number of parameters considered for model training (see supplementary methods). This systematic approach resulted in a total of 35 models belonging to one of three categories: ‘cor models’, ‘Imp models’, and ‘RFE models’ (see Supplementary Fig. S1). Interestingly, a trend towards the selection of parameters from the ‘Advanced’ and ‘Lipoprint’ profiles as the most relevant for distinguishing FH+ from FH− subjects (Supplementary Data S2) was observed. Considering the relatively small size of the corresponding data subsets, we decided to investigate whether it could be influencing the perceived contribution of ‘Advanced’ and ‘Lipoprint’ parameters in our models.

For this purpose, we repeated our analysis (Supplementary Fig. S1) using the biochemical parameters available for each data subset restricting the number of individuals to 78. This number corresponds to the smaller sized subset used in this study (the ‘All’ subset), which comprises the subjects that present measures for all biochemical parameters. Two different approaches were followed: train all the models with the same 78 subjects from the ‘All’ subset; or use a random selection of 78 subjects. This analysis confirmed that parameters from the ‘Advanced’ and ‘Lipoprint’ profiles contribute to a better discrimination between FH+ and FH− status independently of the training set (Supplementary Data S2).

Through careful inspection of all models regarding variable importance and correlation, we noticed that a group of four parameters (LDL1, apoC-III, TC/HDL-C and sdLDL.Day) consistently appeared as highly relevant for the discrimination between FH+ and FH− individuals. However, none of the trained models used this small group of parameters as the only predictors. Such models could be relevant for clinical purposes given their comparative simplicity. Therefore, we decided to train two additional models including only these selected parameters (Sel1 and Sel2, Supplementary Table S3). Given that BMI and age are likely to influence the lipid profile of subjects^12,24^, we further conjugated these parameters with them (models Sel3 and Sel4, Supplementary Table S3). Given the fact that these ‘selected models’ comprise parameters from different lipid profiles, they were trained on the ‘All’ subset.

**Figure 2:**
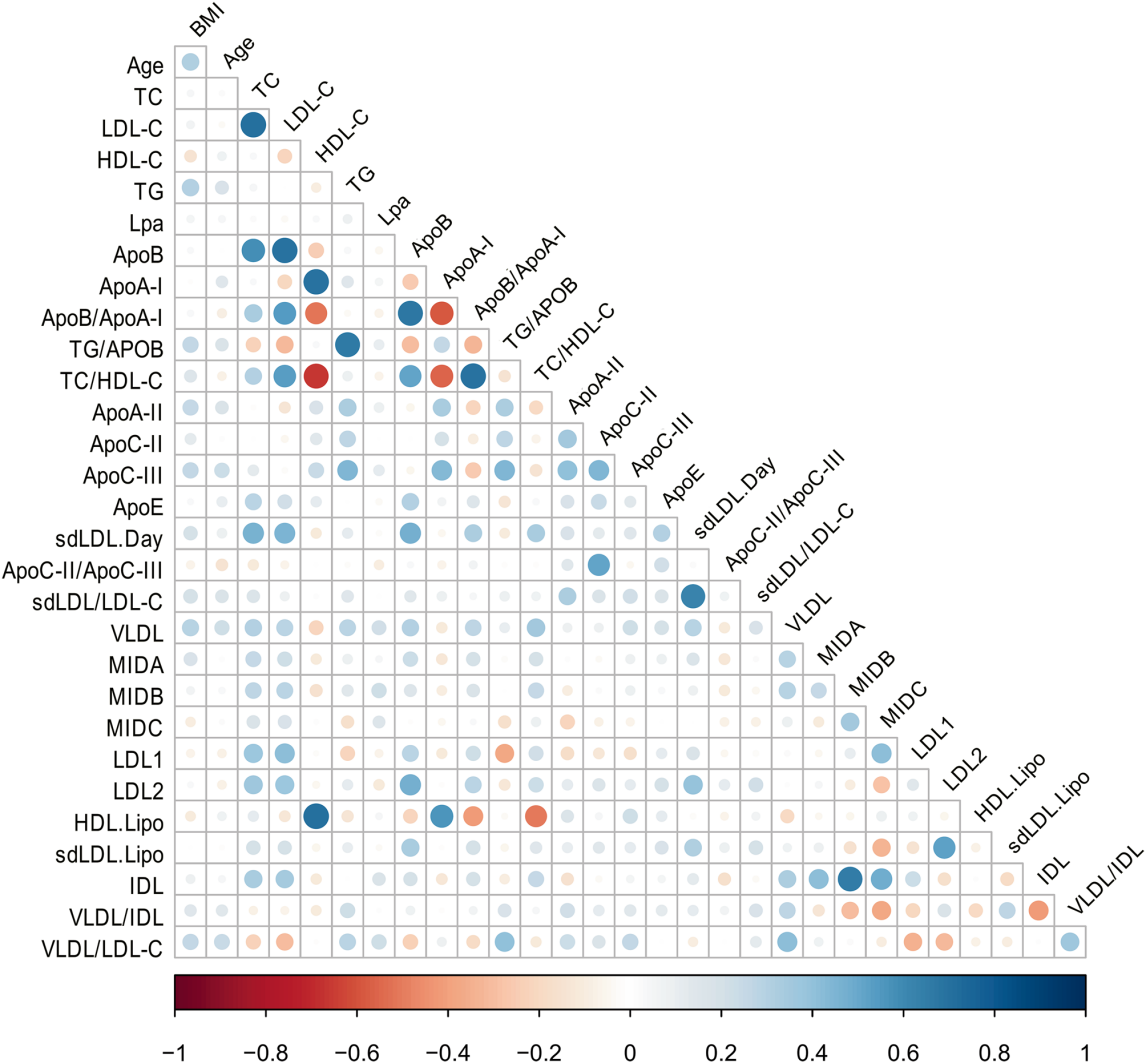
Correlation plot for the dataset parameters. Negative and positive correlations are presented in red and blue, with darker colours corresponding to higher absolute values, according to the scale.

Altogether, a total of 67 models were generated during this analysis (Supplementary Data S2). Given that the presence of models with highly correlated parameters does not contribute substantially to new insights into the biological background of dyslipidaemia, we identified all models containing any pair of parameters whose correlation was equal to or higher than |0.6|. For this purpose, we generated a correlation plot for all parameters used during modelling analysis (Fig. 2). A total of 14 pairs of highly correlated parameters were identified, 12 of which belong to the ‘Basic’ profile. These pairs were found in 32 out of 67 trained models and were thus discarded from further analysis.

### Extended lipid profiles contribute to an improved distinction between FH+ and FH− subjects

Following model training, testing datasets were used to assess model performance and corresponding descriptive statistics were determined. We established a set of ranking criteria to apply to the 35 final models, with cut-off values defined considering the properties and observed range for each statistic (see supplementary methods). We used this approach to retain only the top 10 models (Table 2).

**Table 2:** Top ranking models and performance.

Model	Subset	N	Np	Parameters	Acc	k	Sens	Spec	TP	FN	FP	TN	AUC
Imp_B	Basic	211	3	LDL-C + ApoB/ApoA-I + TG/ApoB	0.84	0.67	0.91	0.86	32	3	7	42	0.92
RFEct_BL	Basic & Lipoprint	95	4	TG/ApoB + TC/HDL-C + TC + LDL1	0.84	0.64	0.83	0.92	10	2	2	23	0.91
Sel3	All	78	5	LDL1 + ApoC-III + TC/HDL-C + BMI + Age	0.77	0.49	0.82	0.90	9	2	2	18	0.89
RFEct_A	All	78	5	LDL1 + TC + ApoA-II + MIDC + TC/HDL-C	0.77	0.46	0.82	0.80	9	2	4	16	0.88
RFE78ct_BL	Basic & Lipoprint	78	5	TC + TC/HDL-C + MIDB + MIDC + LDL1	0.74	0.41	0.82	0.85	9	2	3	17	0.88
RFE78t_B	Basic	78	2	LDL-C + ApoB/ApoA-I	0.81	0.59	0.82	0.85	9	2	3	17	0.87
Sel1	All	78	3	LDL1 + ApoC-III + TC/HDL-C	0.77	0.47	0.82	0.90	9	2	2	18	0.87
Imp_AdL	Advance & Lipoprint	78	3	ApoA-II + ApoC-III + LDL1	0.77	0.47	0.73	0.75	8	3	5	15	0.76
RFE78t_Ad	Advanced	78	5	ApoA-II + ApoC-II + ApoC-III + sdLDL.Day + BMI	0.77	0.49	0.91	0.60	10	1	8	12	0.75
RFE78ct_Ad	Advanced	78	5	Age + ApoA-II + ApoC-II + ApoC-III + sdLDL.Day	0.85	0.66	0.73	0.65	8	3	7	13	0.75

*N* number of individuals, *Np* number of parameters, *Acc* accuracy, *k* Cohen’s kappa coefficient, *Sens* sensitivity, *Spec* specificity, *TP* number of true positives, *FN* number of false negatives, *FP* number of false positives, *TN* number of true negatives, *AUC* area under the ROC curve.

The two best ranked models were the Imp_B and RFEct_BL models, trained with the ‘Basic’ and the ‘Basic & Lipoprint’ subsets, respectively. Among the top 10, these models presented the highest AUC values combined with the best k metrics (Table 2), revealing a substantial agreement between observed and predicted classification of subjects^25^. These models further displayed the best association between sensitivity and specificity, with Imp_B performing better for sensitivity and RFEct_BL for specificity. Of note, eight of the top 10 models were trained using at least one parameter of the ‘Advanced’ and/or ‘Lipoprint’ profiles. The Lipoprint measurement for LDL1 is present in six of these models. The other models (RFE78t_Ad and RFE78ct_Ad) include sdLDL.Day, ApoA-II, ApoC-II and ApoC-III values from the ‘Advanced’ profile. The models that were trained using only parameters from the ‘Basic’ profile include the ApoB/ApoA-I ratio in addition to LDL-C (Imp_B and RFE78t_B). The Imp_B model further includes the TG/ApoB ratio.

In summary, the comparative analysis of model performance revealed that the integration of lipid parameters from different profiles through machine learning can support a robust discrimination between FH+ and FH− subjects (Table 2). Moreover, our results suggest that biochemical parameters not commonly used in clinical practice, but available commercially, may provide important information towards this distinction, namely contributing to a higher specificity.

### Modelling of TC and LDL-C levels improves identification of FH+ individuals in comparison to clinical cut-offs

The biochemical parameters and cut-offs of the SB criteria—defined as blood TC values ≥ 260 mg/ml or LDL-C values ≥ 155 mg/ml for children—are widely used to identify candidate FH individuals and refer them for therapy and genetic testing^12^. Of note, only ~ 60% of the PFHS-ped individuals that fulfilled these criteria were actually FH+, whereas 3 FH+ individuals were found among the 67 that had TC or LDL-C values below these cut-offs.

Given that the SB criteria are based on two simple biochemical parameters, we decided to train two models exclusively using TC and LDL-C and assess their ability to correctly distinguish between FH+ and FH− individuals (‘SB models’, Table 3). These models were trained using all the PFHS-ped subjects or just the ‘Basic & Lipoprint’ subset (Table 2). The resulting models had a weaker performance when compared to top 10 models trained on the same subsets (cf Tables 2 and 3). To explore the differences between SB models and the two best ranked models, we used them to classify 50 individuals randomly selected from the ‘Basic & Lipoprint’ subset (Supplementary Table S4). Specificity, sensitivity and the positive and negative predictive values (PPV and NPV, respectively) were calculated for the predictions made by these models, as well as for the FH+/FH− classification according to SB criteria cut-offs (Supplementary Table S4). As expected, SB criteria have a very high sensitivity and NPV. However, they are extremely unspecific, with a high likelihood of selection of FH− patients for genetic testing. SB models can considerably improve on this, although they present a lower sensitivity in comparison to SB cut-offs. However, in contrast with SB cut-offs, these models present a very good balance between sensitivity and specificity (Supplementary Table S4). The two top-ranked models trained with the extended lipid profile can achieve very good PPVs while keeping acceptable values for sensitivity and NPV.

**Table 3:** Performance of models trained with SB criteria parameters.

Model	Subset	N	Np	Parameters	Acc	k	Sens	Spec	TP	FN	FP	TN	AUC
SB_B	Basic	211	2	TC + LDL-C	0.80	0.57	0.77	0.82	27	8	9	40	0.89
SB_BL	Basic & Lipoprint	95	2	TC + LDL-C	0.81	0.56	0.67	0.84	8	4	4	21	0.84

Column names as defined in Table 2 legend.

These results emphasize how modelling approaches can improve patient classification compared to the use of strict cut-off values. The reduced performance of SB models in comparison to top 10 models supports our suggestion that extended lipid parameters contain relevant biological information for an improved classification of FH+ and FH− individuals.

### Implementing the best-ranking models in a clinical setting

Our top 10 models can be easily used in clinical practice to prioritize patients for genetic testing. Clinicians can access the different models and select the one that better suits their practice, in the following link: https://github.com/GamaPintoLab/FH-Models-.git. Models can be grouped into three different categories, depending on the availability of parameters required to run them. A first set of models, including the best ranked model, require biochemical parameters that can be provided by most clinical laboratories. Other models include additional values for ApoA-II, ApoC-II, ApoC-III, sdLDL.Day, which are only available in more specialized clinical laboratories, while the final set of models relies on ‘Lipoprint’ parameters LDL1, MIDC or MIDB, a method that is currently for research use only. We provide an Excel file for simple implementation of the two best ranked models (Table 2) and the SB_B model, which classifies patients as FH+ or FH− upon introduction of the required parameter values. In addition, all top 10 models can be downloaded and applied to a new dataset using R software.

## Discussion

Given the high risk for severe CVD at an early age and the benefits of early therapeutic intervention, the identification of children carrying monogenic FH mutations is of extreme importance. Biochemical identification of dyslipidaemic subjects in clinical practice usually relies on the analysis of serum levels for total cholesterol, HDL-C, TG, LDL-C and eventually apoA-I and apoB^11,26^. Although these biochemical markers allow for a relatively sensitive screening of individuals at risk for CVD, including FH candidates, their specificity in distinguishing monogenic individuals is very low^27^. In addition, several studies show that many children do not comply with multiple parameters of clinical diagnostic criteria, including the presence of family history of hypercholesterolaemia/CVD or LDL-C levels above the defined cut-offs^9,11^. Screening for genetic mutations was therefore recommended as standard of care for patients with definite or probable FH by an international Expert Consensus Panel^9^. However, the diagnostic yield of these screening programs is low^28^, ranging between 20 and 80%^29^, as a high number of suspected patients suffer from polygenic conditions^9^. Thus, the development of robust approaches that can contribute to increase this yield is critical to support a widespread use of FH genetic testing, with a considerable reduction of the resulting burden on health systems.

In this study, we have applied machine learning-based methods to perform a thorough analysis of the extended lipid profiles of the PFHS-ped dataset. We hypothesized that using an extended lipid profile would confer an additional layer of information, supporting a more accurate identification of FH+ subjects, leading to the identification of novel clinically relevant biomarkers. Multiple ‘training’ sets comprising different combinations of biochemical parameters were used to train classification models to distinguish FH+ and FH− individuals, followed by an assessment of performance on independent ‘testing’ sets. For comparison purposes, similar models using only TC and LDL-C were trained. Predictions of FH+ and FH− status for the same group of patients were performed using the two best models, SB models and standard SB criteria cut-offs (Supplementary Table S4). Results show that modelling can considerably improve the specific identification of FH+ individuals and the PPV, with a limited impact on the high sensitivity afforded by SB cut-off criteria. Furthermore, the inclusion of extended lipid parameters contributes to an improved patient identification.

The best ranking model Imp_B uses ApoB/ApoA-I and TG/ApoB ratios, in addition to LDL-C levels, to generate predictions with the highest sensitivity values. Of note, LDL-C levels used in this study were directly determined and thus their accuracy is not affected by TG levels. The current guidelines for dyslipidaemia already recommend the determination of LDL-C, TG and apoB in all dyslipidaemic individuals^26^. Like the TC/HDL-C, the ApoB/ApoA-I ratio has been linked to cardiovascular risk^30^. Indeed, a previous study identified the ApoB/ApoA-I ratio as a potential biomarker for FH^12^. The TG/ApoB ratio was selected both in the first and second ranked models, the later delivering the highest specificity and PPV. This model further includes two ‘Basic’ biochemical parameters (TC and TC/HDL-C) and LDL1 from Lipoprint analysis (see methods). Of note, LDL1 is the most commonly selected biochemical parameter across all top 10 models, suggesting it holds relevant information for the specific identification of FH+ individuals.

The parameters used by the best two models are in agreement with the biology behind FH. Supplementary Fig. 2 shows data for these parameters. TC and LDL-C have higher values for FH+ compared to FH− subjects. This is unsurprising, because FH+ subjects present single-gene mutations that disrupt the clearance of LDL particles by the liver^31^. The TG/ApoB ratio is lower for FH+ compared to FH− subjects. This is understandable, given both the lower clearance of ApoB in FH+ subjects as well as a higher expected TG in FH− subjects. Hypercholesterolaemia in FH− subjects is likely to have environmental influence, such as cholesterol and TG-rich diets. This should lead to a production of more and ‘bigger’ VLDL particles, containing more TG^32^. It has been shown that fatty acids can also modulate lipoprotein lipolysis and clearance^33^. Therefore, the observed TG/ApoB ratio difference is biologically understandable. The TC/HDL-C and ApoB/ApoA-I ratios are higher for FH+ compared to FH− subjects. Higher TG availability in FH− subjects leads to more lipolysis of VLDL through LPL. The cholesterol released is transported back to the liver as HDL, raising HDL-C and ApoA-I concentrations. This mechanism is plausible because LPL gain-of-function and loss-of-function polymorphisms lead to higher and lower HDL-C respectively^34^. We consistently find higher LDL1 concentration for FH+ versus FH− subjects. This is in accordance with the findings of Teng et al.^35^. Explaining the observed high LDL1 requires distinguishing between lipolysis through lipoprotein lipase (LPL) and hepatic lipase (HL). The mechanistic modelling study by van Schalkwijk et al.^36^ suggests that lipolysis outside the liver by LPL mostly affects larger ApoB-containing lipoproteins such as VLDL, while HL mostly targets smaller IDL through LDL particles. Given the impaired binding of ApoB-containing particles to LDLR on the liver, FH+ subjects can be expected to have a lower HL lipolysis and liver clearance than FH− subjects. The lower HL lipolysis explains the accumulation of LDL1 particles. Therefore, even though other LDL subfractions will increase due to a longer circulation time, accumulation of the larger LDL1 particles is especially marked. In addition, several studies have associated altered HL activity or expression, namely in association to genetic polymorphisms, to more severe FH phenotypes^37,38^. All parameters identified in this study to discriminate between FH+ and FH− subjects are therefore biologically plausible.

Overall, our results suggest that modelling, together with the inclusion of novel lipid parameters, can support an improved classification of FH+ and FH− individuals, with a significant impact on the yield of genetic screening programs and corresponding costs.

Our top models can already be used by clinicians to obtain more precise estimates of the likelihood that their patients are FH+ in comparison to SB criteria. The PPVs and NPVs described in Supplementary Table S4 should be taken into consideration when interpreting results. All the required information for their application is provided in GitHub (see link in Results). The availability of larger patient datasets will be crucial to identify which of the new, non-standard parameters used by our models will be worth incorporating into clinical practice.

## Supplementary Information

Supplementary Information 1.

Supplementary Information 2.

Supplementary Information 3.
