# Supplementary material for: Machine learning modelling of blood lipid biomarkers in familial hypercholesterolaemia versus polygenic/environmental dyslipidaemia
Source: Sci Rep. 2021 Feb 15;11:3801. doi: 10.1038/s41598-021-83392-w (PMC7884847; doi:10.1038/s41598-021-83392-w)
Supplement: Supplementary file 1 — Supplementary Information 1. [file 41598_2021_83392_MOESM1_ESM.pdf]

Machine learning modelling of blood lipid biomarkers in familial hypercholesterolaemia versus polygenic/environmental dyslipidaemia

Marta Correia[1,3], Eva Kagenaar[2], Daniël Bernardus van Schalkwijk[2], Mafalda Bourbon[1,3*], Margarida Gama-Carvalho[1*†]

[1] University of Lisboa, Faculty of Sciences, BioISI – Biosystems & Integrative Sciences Institute, Campo Grande, 1749-016 Lisboa, Portugal

[2] Amsterdam University College, Science Park 113, 1098 XG Amsterdam, Netherlands

[3] National Institute of Health Doutor Ricardo Jorge, Padre Cruz Av., 1649-016 Lisboa, Portugal

[*]Co-senior authors

[†] Corresponding author (e-mail: mhcarvalho@fc.ul.pt)

Supplementary figures and tables

Supplementary methods

SUPPLEMENTARY FIGURES

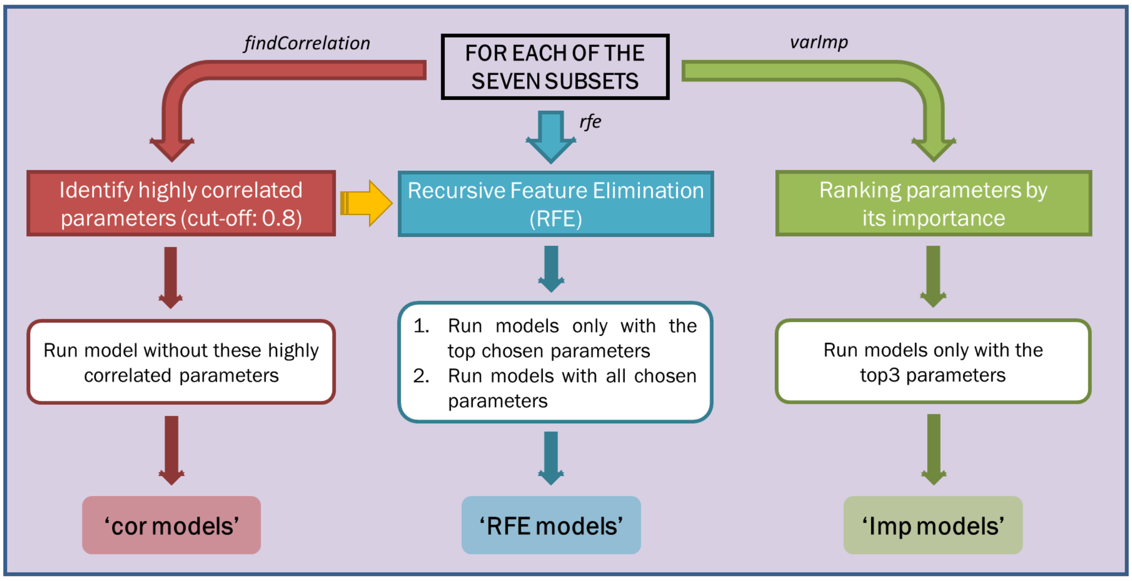

Figure S1. Modelling workflow using three methods to avoid overfitting, producing three groups of models: cor, RFE, and Imp.

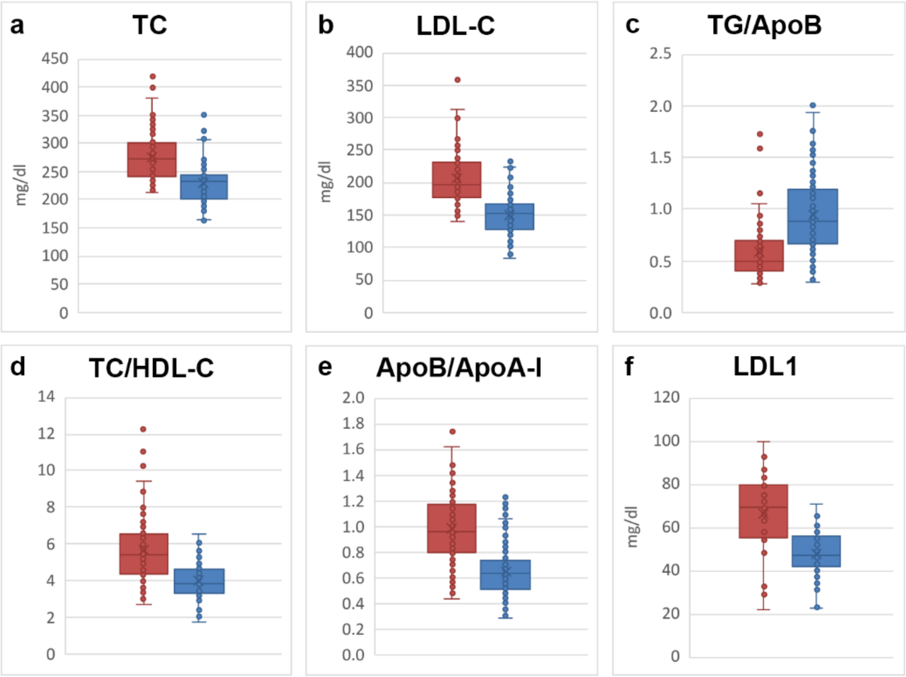

Figure S2. Box and whiskers plots with distribution of individual values for the parameters used by the two top ranking models according to patient classification as FH+ (red) or FH- (blue). a) total cholesterol (TC); b) LDL-cholesterol (LDL-C); c) ratio between triglycerides and apolipoprotein B (TG/ApoB); d) ratio between total cholesterol and HDL-cholesterol (TC/HDL-C); e) ratio between apolipoprotein B and apolipoprotein A-I (ApoB/apoA-I); f) buoyant (large) LDL fraction 1 (LDL1). All individuals with measured TC, LDL-C, and LDL1 were used for the plots a), b) and f), respectively. For ratios, all individuals with measurements for both parameters were used.

SUPPLEMENTARY TABLES

Table S1. Mean value of each biochemical parameter by subset, for FH+ individuals. Grey cells correspond to the absence of that parameter in the given subset. sdLDL.Day and sdLDL.Lipo were measured by different techniques, using RX daytona+® analyser and Lipoprint® assay, respectively.

Subsets / Parameters	All	Basic	Advanced	Lipoprint	Basic & Advanced	Basic & Lipoprint	Advanced & Lipoprint
TC	271.32	274.87			270.37	274.47	
LDL-C	203.25	205.66			202.32	205.50	
HDL-C	49.96	51.72			50.42	50.66	
TG	72.86	74.73			71.11	74.09	
Lpa	45.21	40.32			43.01	41.94	
ApoB	129.61	129.24			130.63	130.19	
ApoA-I	133.18	135.35			133.47	135.09	
ApoB/ApoA-I	1.01	0.99			1.02	1.00	
TG/ApoB	0.57	0.59			0.56	0.58	
TC/HDL-C	5.74	5.68			5.69	5.75	
ApoA-II	27.54		26.55		26.55		27.54
ApoC-II	3.10		2.94		2.94		3.10
ApoC-III	6.95		6.66		6.66		6.95
ApoE	3.81		3.69		3.69		3.81
sdLDL.Day	41.47		38.44		38.44		41.47
ApoC-II/ApoC-III	0.43		0.43		0.43		0.43
sdLDL/LDL-C	0.20		0.19		0.19		0.20
VLDL	34.39			34.47		34.47	34.39
MIDA	26.46			27.88		27.88	26.46
MIDB	19.71			20.16		20.16	19.71
MIDC	29.18			28.91		28.91	29.18
LDL1	65.07			66.59		66.59	65.07
LDL2	31.96			32.00		32.00	31.96
HDL.Lipo	53.14			53.69		53.69	53.14
sdLDL.Lipo	5.43			5.63		5.63	5.43
IDL	75.36			76.94		76.94	75.36
VLDL/IDL	0.47			0.46		0.46	0.47
VLDL/LDL-C	0.17			0.17		0.17	0.17

Table S2. Mean value of each biochemical parameter by subset, for FH- individuals. Grey cells correspond to the absence of that parameter in the given subset. sdLDL.Day and sdLDL.Lipo were measured by different techniques, using RX daytona+® analyser and Lipoprint® assay, respectively.

Parameters \ Subsets	All	Basic	Advanced	Lipoprint	Basic & Advanced	Basic & Lipoprint	Advanced & Lipoprint
TC	230.46	228.49			232.36	232.02	
LDL-C	152.96	150.87			155.19	154.86	
HDL-C	60.56	60.11			60.36	59.73	
TG	96.66	93.64			92.78	97.94	
Lpa	45.74	54.89			48.93	49.84	
ApoB	96.96	99.33			99.27	98.63	
ApoA-I	164.08	156.69			161.93	162.25	
ApoB/ApoA-I	0.61	0.65			0.64	0.63	
TG/ApoB	1.00	0.95			0.94	0.98	
TC/HDL-C	4.02	3.99			4.06	4.08	
ApoA-II	31.37		30.44		30.44		31.37
ApoC-II	4.52		4.34		4.34		4.52
ApoC-III	9.09		8.82		8.82		9.09
ApoE	3.45		3.37		3.37		3.45
sdLDL.Day	30.97		30.52		30.52		30.97
ApoC-II/ApoC-III	0.51		0.50		0.50		0.51
sdLDL/LDL-C	0.20		0.19		0.19		0.20
VLDL	29.80			30.86		30.86	29.80
MIDA	22.52			23.62		23.62	22.52
MIDB	16.36			17.38		17.38	16.36
MIDC	21.28			21.97		21.97	21.28
LDL1	48.04			47.63		47.63	48.04
LDL2	26.16			24.86		24.86	26.16
HDL.Lipo	57.60			56.94		56.94	57.60
sdLDL.Lipo	7.46			7.67		7.67	7.46
IDL	60.16			62.97		62.97	60.16
VLDL/IDL	0.51			0.51		0.51	0.51
VLDL/LDL-C	0.20			0.20		0.20	0.20

Table S3. Identification of the manually selected parameters that comprise each of the four 'selected models'. N: number of individuals; Np: number of parameters.

Model	N	Np	Parameters
Sel1	78	3	LDL1 + ApoC-III + TC/HDL-C
Sel2	78	4	LDL1 + ApoC-III + TC/HDL-C + sdLDL.Day
Sel3	78	5	LDL1 + ApoC-III + TC/HDL-C + BMI + Age
Sel4	78	6	LDL1 + ApoC-III + TC/HDL-C + sdLDL.Day + BMI + Age

Table S4. Comparison of classification performance between the best two ranked models, 'SB models' and SB criteria for the same universe of 50 individuals (randomly selected from 'Basic & Lipoprint' subset). PPV: positive predictive value; NPV: negative predictive value.

		Specificity	Sensitivity	PPV	NPV
**Models**	Imp_B	0.89	0.93	0.78	0.97
	RFEct_BL	0.97	0.87	0.93	0.94
	SB_BL	0.83	0.73	0.65	0.88
	SB_B	0.86	0.80	0.71	0.91
**SB criteria**		0.49	1	0.45	1

SUPPLEMENTARY METHODS

Modelling and data analysis

All the analysis was developed using R software (version 3.4.3) [1]. The *caret* package for machine learning [2] was used to train classification models based on logistic regression, and a resampling scheme of three times cross validation was applied to estimate model accuracy. Accordingly, data was randomly divided in two sets of 60% and 40% of the subjects defining the training and the testing sets, respectively. The training set was used for model generation and the testing set was used for posterior validation. The Bayesian generalized linear model (*bayesglm*) was applied on the training set using *train* function of *caret* package [3]. To avoid overfitting the number of parameters considered for model training was reduced using three different methods available in *caret* : 1) exclusion of one element of a pair of highly correlated parameters (cut-off = 0.8; 'cor models'); 2) ranking of parameters by importance based on a ROC analysis of each parameter with only the top 3 variables selected for model training ('Imp models'); 3) recursive feature elimination (RFE) of parameters ('RFE models'); when more than five parameters were selected by RFE, models were trained both with the top 5 and with all parameters. Additional 'RFE models' were generated after removal of highly correlated parameters. To identify highly correlated parameters, *cor* function was applied using Kendal's tau method for mixed and tied data [4]. Following model training, their predictive performance was assessed on the testing set. The *predict* function of *caret* was used along with *confusionMatrix* to acquire the main statistics regarding model performance, which were organized in a table using *broom* package [5]. The *pROC* package [6] was used to measure the area under the ROC curve (AUC) regarding individuals classification.

Model ranking criteria were defined as follows: 1) sorting by AUC (highest to lowest) reflects a better performance regarding the relation between specificity and sensitivity; 2) k values above 0.4 correspond to a moderate agreement between observed and predicted classes [7]; 3) reduced number of parameters (≤5) for model simplicity; and 4) highest sensitivity (≥0.7) cut-off values. Criteria were applied in that order to generate a ranked list of the best models, 11 in total. The Akaike information criterion (AIC) was used to eliminate one model to restrict the final list to a 'top 10'.

<u>*References*</u>

[1] R Core Team, "R: A language and environment for statistical computing," *R Foundation for Statistical Computing*. Vienna, 2017.

[2] M. K. C. from Jed Wing *et al.*, "caret: Classification and Regression Training." 2018.

[3] A. Gelman and Y.-S. Su, "arm: Data Analysis Using Regression and Multilevel/Hierarchical Models." 2018.

[4] A. J. Bishara and J. B. Hittner, "Testing the significance of a correlation with nonnormal

data: Comparison of Pearson, Spearman, transformation, and resampling approaches.," *Psychol Methods*, vol. 17, no. 3, pp. 399–417, 2012.
[5] D. Robinson and A. Hayes, "broom: Convert Statistical Analysis Objects into Tidy Tibbles." 2018.
[6] X. Robin *et al.*, "pROC: an open-source package for R and S+ to analyze and compare ROC curves," *BMC Bioinformatics*, vol. 12, p. 77, 2011.
[7] J. R. Landis and G. G. Koch, "The Measurement of Observer Agreement for Categorical Data," *Biometrics*, vol. 33, no. 1, pp. 159–174, 1977.
